# Supplementary material for: Prognostic Impact of Low Skeletal Muscle Mass on Major Adverse Cardiovascular Events in Coronary Artery Disease: A Propensity Score-Matched Analysis of a Single Center All-Comer Cohort
Source: J Clin Med. 2019 May 19;8(5):712. doi: 10.3390/jcm8050712 (PMC6571815; doi:10.3390/jcm8050712)
Supplement: Supplementary file 1 [file jcm-08-00712-s001.pdf]

Prognostic impact of low skeletal muscle mass on major adverse cardiovascular events in coronary artery disease: a propensity score-matched analysis of single center all-comer cohort

SUPPLEMENTAL FIGURE LEGENDS

Figure S1. Flow chart of patient enrollment.
CT = computed tomography; L1 = first lumbar vertebra; PCI = percutaneous coronary intervention; PSM = propensity-score matched; SMA = skeletal muscle area; SMI = skeletal muscle index

Figure S2. Receiver operating characteristic curves generated for 3-year clinical outcomes based on L1 SMI. Results for the overall study population (A, D) and sex-specific results for the males (B, E) and the females (C, F) are displayed.
AUC = area under the curve; CI = confidence interval; L1 = first lumbar vertebra; MACE = major adverse cardiovascular event; SMI = skeletal muscle index

Figure S3. Correlation between BMI and measured L1 SMI.
BMI = body mass index; L1 = first lumbar vertebra; SMI = skeletal muscle index

Figure S4. Intra- and Interobserver variability of L1 SMI measurement. Intraobserver agreement of observer 1 (A, B) and observer 2 (C, D) with interobserver agreement between observer 1 and observer 2 (E, F) are displayed.
L1 = first lumbar vertebra; ICC = intra-class correlation coefficient; SMI = skeletal muscle index

Figure S1. Flow chart of patient enrollment.

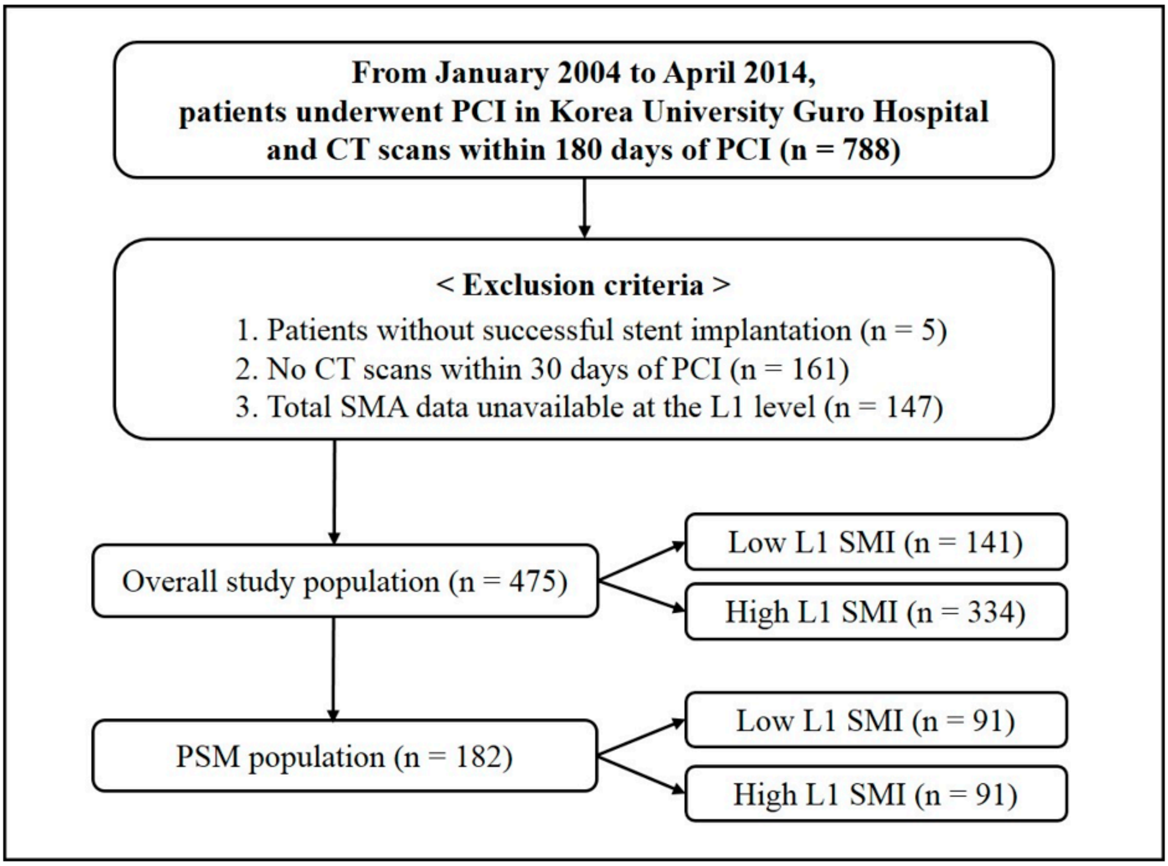

Figure S2. Receiver operating characteristic curves generated for 3-year clinical outcomes based on L1 SMI.

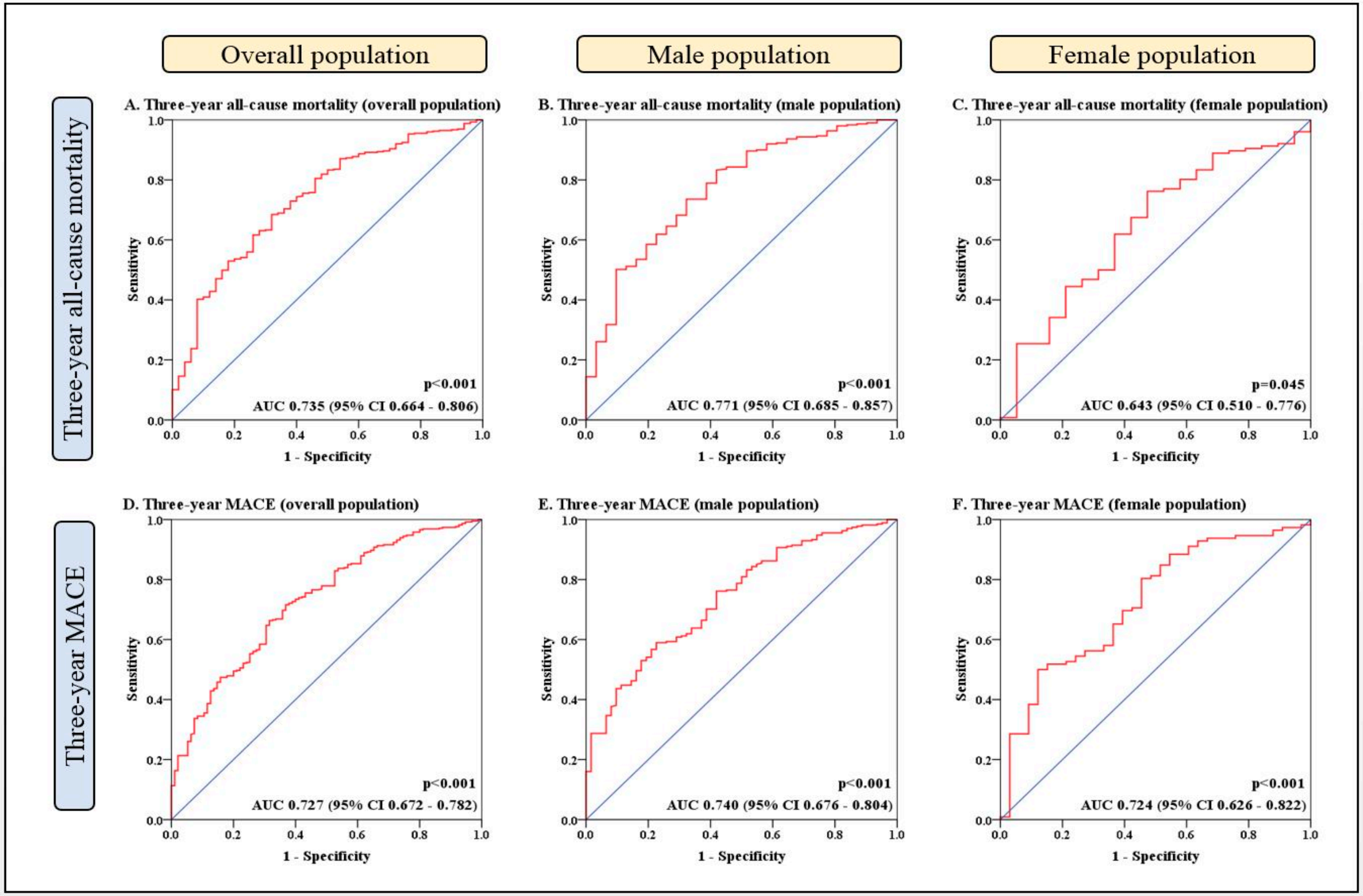

Figure S3. Correlation between BMI and measured L1 SMI.

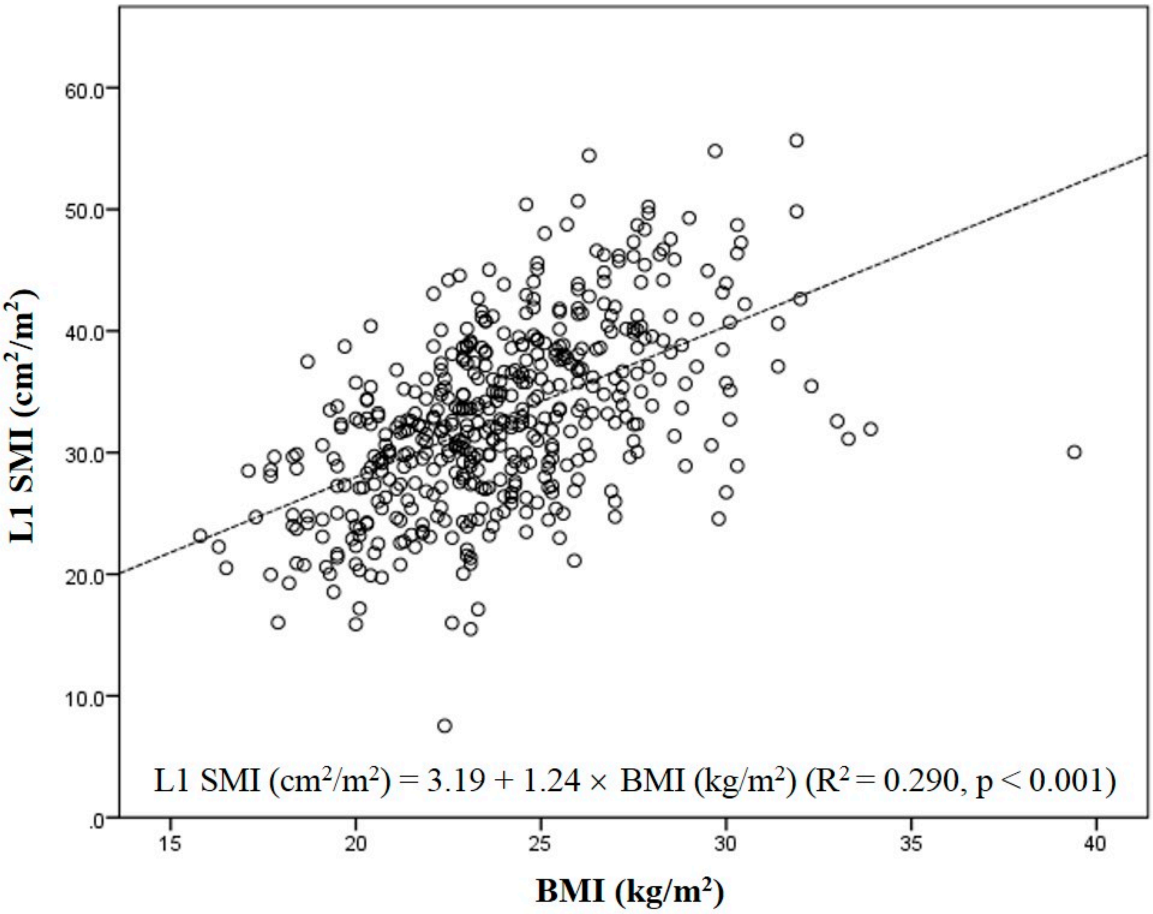

Figure S4. Intra- and Interobserver variability of L1 SMI measurement.

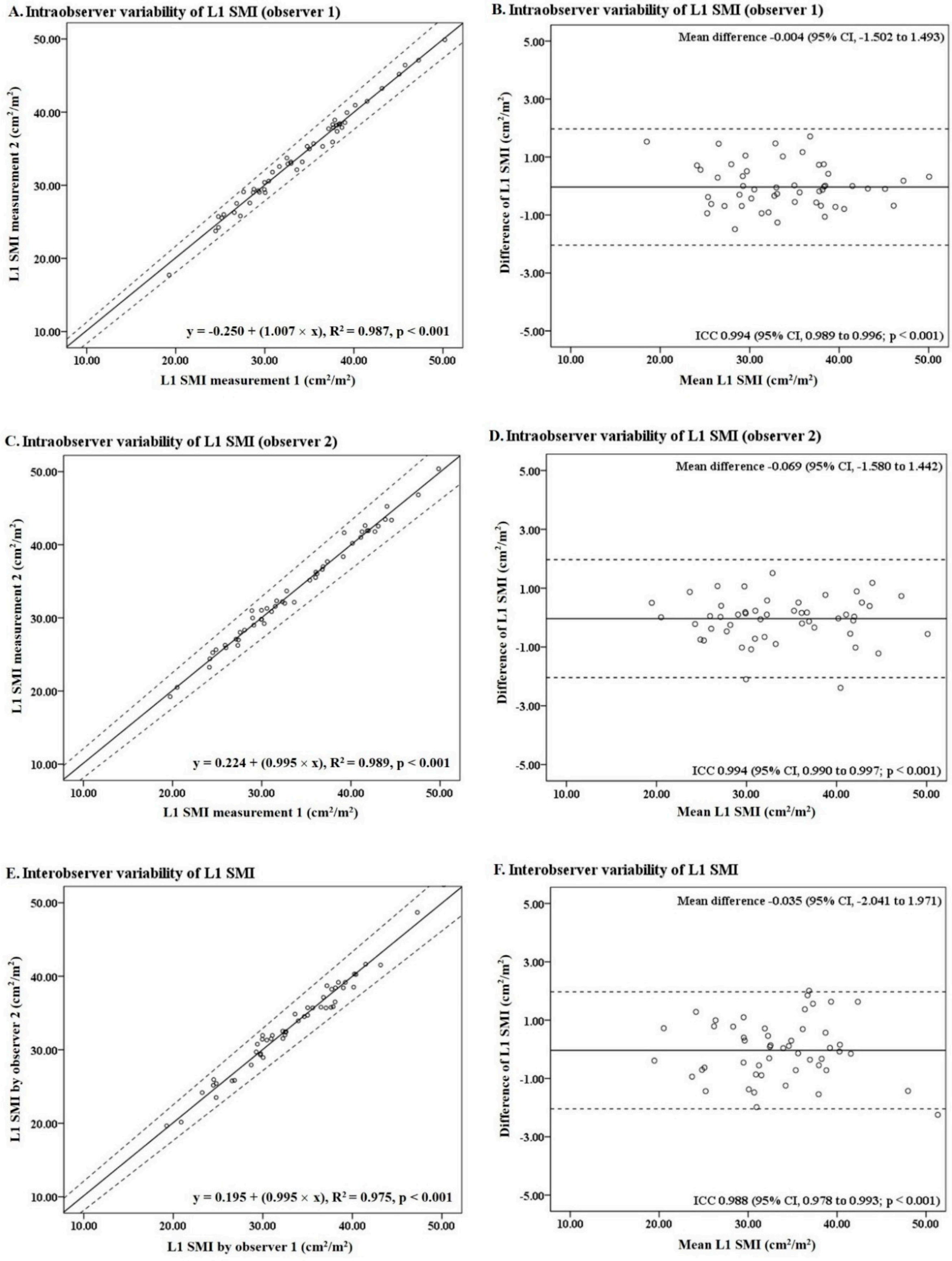

Table S1. Detailed characteristics of performed CT scans

	Overall population				PSM population			
	Low L1 SMI (n = 141)	High L1 SMI (n = 334)	p-value	S.diff	Low L1 SMI (n = 91)	High L1 SMI (n = 91)	p-value	S.diff
**Modality of CT scan**								
Chest CT	36 (25.5)	68 (20.3)	0.043	-0.02	23 (25.2)	23 (25.2)	0.72	0.06
Coronary CT	16 (11.3)	74 (22.1)			13 (14.2)	14 (15.3)		
Abdomen CT	43 (30.4)	77 (23.0)			24 (26.3)	17 (18.6)		
Lower extremity CT angiography	41 (29.0)	99 (29.6)			29 (31.8)	33 (36.2)		
Torso PET-CT	5 (3.5)	16 (4.7)			2 (2.1)	4 (4.3)		
**Purpose of CT scan**								
Elective diagnostic exam	70 (49.6)	198 (59.2)	0.05	0.22	44 (48.3)	42 (46.1)	0.77	-0.05
Emergency examination	71 (50.3)	136 (40.7)			47 (51.6)	49 (53.8)		

Data are expressed as n (%). CT = computed tomography; L1 = first lumbar vertebra; PET = positron emission tomography; PSM = propensity score-matched; S.diff = standardized difference; SMI = skeletal muscle index

Table S2. Specific cause of non-cardiovascular death

	Low L1 SMI (n = 141)	High L1 SMI (n = 334)
Overall non-cardiovascular deaths	21 (100.0)	12 (100.0)
Infection	11 (52.3)	5 (41.6)
Fatal bleeding*	5 (23.8)	3 (25.0)
Respiratory disease	3 (14.2)	2 (16.6)
Renal failure	1 (4.7)	0 (0.0)
Others	1 (4.7)	2 (16.6)

Data are expressed as n (%). *Fatal bleeding included deaths from intracranial hemorrhage and gastrointestinal bleeding. L1 = first lumbar vertebral; SMI = skeletal muscle index

Table S3. Complete dataset of univariate analysis and stepwise multivariate analysis for 3-year clinical outcomes

A. Univariate analysis

	Original population			
	3-year all-cause mortality		3-year MACE	
	HR (95% CI)	p-value	HR (95% CI)	p-value
Low L1 SMI	4.34 (2.45-7.69)	<0.001	3.82 (2.54-5.74)	<0.001
Sex (male)	0.70 (0.39-1.23)	0.21	0.79 (0.51-1.20)	0.26
Age >65 years	5.95 (2.53-13.96)	<0.001	2.13 (1.36-3.32)	0.001
BMI (per 1.0 kg/m^2 increase)	0.91 (0.83-0.99)	0.048	0.85 (0.89-1.02)	0.15
LVEF <50%	1.95 (1.10-3.48)	0.023	1.78 (1.16-2.74)	0.008
CrCl <60 mL/min	3.57 (2.02-6.29)	<0.001	2.82 (1.89-4.22)	<0.001
Total cholesterol >5.17 mmol/L (>200 mg/dL)	0.33 (0.12-0.92)	0.034	0.59 (0.33-1.06)	0.08
Clopidogrel at discharge	0.12 (0.07-0.20)	<0.001	0.27 (0.17-0.42)	<0.001
Statins at discharge	0.14 (0.08-0.24)	<0.001	0.29 (0.19-0.44)	<0.001
Average stent diameter (per 1.0 mm increase)	1.14 (0.60-2.17)	0.70	0.70 (0.43-1.15)	0.16
Clinical presentation as myocardial infarction	1.48 (0.83-2.62)	0.18	1.22 (0.79-1.87)	0.37
Hypertension	1.28 (0.70-2.34)	0.42	1.15 (0.75-1.76)	0.53
Diabetes	2.64 (1.47-4.73)	0.001	2.08 (1.38-3.13)	<0.001
Previous malignancy	1.93 (0.82-4.53)	0.13	1.31 (0.64-2.70)	0.46
Multivessel disease	1.17 (0.63-2.17)	0.62	1.41 (0.92-2.17)	0.12
Second generation DES	0.75 (0.40-1.42)	0.38	0.62 (0.40-0.96)	0.030
Frailty (CFS ≥5)	3.99 (2.28-6.98)	<0.001	2.07 (1.37-3.13)	<0.001

BMI = body mass index; CI = confidence interval; CFS = Clinical Frailty Scale; CrCl = creatinine clearance; DES = drug eluting stents; L1 = first lumbar vertebra; LVEF = left ventricular ejection fraction; MACE = major adverse cardiovascular event; OR = odds ratio; PCI = percutaneous coronary intervention; SMI = skeletal muscle index

B. Stepwise multivariate analysis

	Original population			
	3-year all-cause mortality		3-year MACE	
	HR (95% CI)	p-value	HR (95% CI)	p-value
Model 2				
Low L1 SMI	3.74 (1.89-7.41)	<0.001	4.51 (2.76-7.38)	<0.001
Sex (male)	0.93 (0.52-1.68)	0.83	0.86 (0.55-1.35)	0.53
Age >65 years	4.39 (1.81-10.6)	0.001	1.51 (0.93-2.46)	0.09
BMI (per 1.0 kg/m^2 increase)	1.03 (0.93-1.13)	0.52	1.07 (1.00-1.14)	0.042
Model 3				
Low L1 SMI	3.69 (1.81-7.52)	<0.001	3.77 (2.27-6.27)	<0.001
Sex (male)	0.80 (0.42-1.52)	0.50	0.90 (0.56-1.45)	0.68
Age >65 years	3.07 (1.23-7.66)	0.016	1.21 (0.73-2.00)	0.45
BMI (per 1.0 kg/m^2 increase)	1.06 (0.96-1.17)	0.21	1.10 (1.02-1.18)	0.007
LVEF <50%	1.51 (0.81-2.80)	0.19	1.28 (0.81-2.00)	0.28
CrCl <60 mL/min	1.48 (0.78-2.79)	0.23	1.75 (1.09-2.79)	0.018
Total cholesterol >5.17 mmol/L (>200 mg/dL)	0.39 (0.13-1.17)	0.10	0.70 (0.37-1.29)	0.26
Clopidogrel at discharge	0.18 (0.09-0.36)	<0.001	0.36 (0.21-0.60)	<0.001
Statins at discharge	0.32 (0.17-0.62)	0.001	0.47 (0.29-0.75)	0.002
Average stent diameter (per 1.0 mm increase)	1.33 (0.68-2.59)	0.40	0.86 (0.51-1.43)	0.57
Model 4				
Low L1 SMI	4.07 (1.95-8.45)	<0.001	3.76 (2.27-6.23)	<0.001
Sex (male)	0.75 (0.39-1.45)	0.40	0.83 (0.51-1.35)	0.48
Age >65 years	2.77 (1.09-7.01)	0.031	1.11 (0.66-1.86)	0.68
BMI (per 1.0 kg/m^2 increase)	1.06 (0.96-1.17)	0.22	1.09 (1.01-1.17)	0.012
LVEF <50%	1.28 (0.63-2.59)	0.48	1.17 (0.71-1.94)	0.52

CrCl <60 mL/min	1.32 (0.69-2.54)	0.40	1.57 (0.97-2.52)	0.06
Total cholesterol >5.17 mmol/L (>200 mg/dL)	0.39 (0.13-1.18)	0.10	0.70 (0.38-1.31)	0.28
Clopidogrel at discharge	0.18 (0.09-0.36)	<0.001	0.36 (0.21-0.62)	<0.001
Statins at discharge	0.33 (0.17-0.66)	0.001	0.50 (0.31-0.81)	0.005
Average stent diameter (per 1.0 mm increase)	1.36 (0.67-2.72)	0.39	0.88 (0.52-1.50)	0.66
Clinical presentation as myocardial infarction	1.43 (0.71-2.88)	0.32	1.23 (0.76-2.01)	0.39
Hypertension	1.44 (0.74-2.80)	0.28	1.01 (0.63-1.61)	0.95
Diabetes	1.48 (0.78-2.81)	0.22	1.48 (0.95-2.31)	0.08
Previous malignancy	1.50 (0.60-3.75)	0.38	1.34 (0.62-2.86)	0.45
Multivessel disease	0.77 (0.39-1.49)	0.44	1.14 (0.72-1.81)	0.55
Second generation DES	0.89 (0.46-1.72)	0.73	0.70 (0.44-1.10)	0.13

BMI = body mass index; CI = confidence interval; CrCl = creatinine clearance; DES = drug eluting stents; L1 = first lumbar vertebra; LVEF = left ventricular ejection fraction; MACE = major adverse cardiovascular event; OR = odds ratio; PCI = percutaneous coronary intervention; SMI = skeletal muscle index

Table S4. Kaplan-Meier survival analysis of 3-year clinical outcomes by the presence of frailty

	Frail (n=121)	Non-frail (n=354)	Log-rank p-value
All-cause mortality	28 (24.8)	22 (6.4)	<0.001
Cardiovascular	10 (9.7)	7 (2.0)	0.001
Non-cardiovascular	18 (16.7)	15 (4.1)	<0.001
Non-fatal MI	7 (7.2)	10 (3.0)	0.10
ST-elevation MI	2 (2.5)	7 (2.1)	0.89
Non-ST-elevation MI	5 (4.8)	3 (1.0)	0.010
Repeat revascularization	10 (11.8)	36 (12.1)	0.89
TVR	9 (10.6)	27 (9.1)	0.73
Non-TVR	1 (1.2)	14 (4.8)	0.14
MACE	37 (33.4)	58 (17.8)	<0.001

Data are expressed as n (%). MACE = major adverse cardiovascular event; MI = myocardial infarction; PSM = propensity-score matched; TVR = target vessel revascularization

Table S5. Multivariate analysis of 3-year clinical outcomes by the presence of frailty

	3-year all-cause mortality		3-year MACE	
	HR (95% CI)	p-value	HR (95% CI)	p-value
Model 1*				
Frailty (CFS ≥5)	3.99 (2.28-6.98)	<0.001	2.07 (1.37-3.13)	<0.001
Model 2†				
Frailty (CFS ≥5)	3.29 (1.87-5.79)	<0.001	1.84 (1.21-2.81)	0.004
Model 3‡				
Frailty (CFS ≥5)	2.46 (1.33-4.55)	0.004	1.37 (0.87-2.15)	0.17
Model 4§				
Frailty (CFS ≥5)	2.81 (1.47-5.39)	0.002	1.29 (0.81-2.05)	0.28

* Univariate analysis; † adjusted for age, sex, and body mass index; ‡ further adjusted for left ventricular ejection fraction <50%, creatinine clearance <60 mL/min, Total cholesterol >5.17 mmol/L (>200 mg/dL), clopidogrel at discharge, statins at discharge, average diameter of inserted stents; § further adjusted for clinical presentation as myocardial infarction, hypertension, diabetes, previous malignancy, multivessel disease, and implantation of 2nd generation drug-eluting stents. CFS = Clinical Frailty Scale; CI = confidence interval; HR = hazard ratio; MACE = major adverse cardiovascular event

Table S6. Baseline characteristics and procedural profiles: comparison between included and excluded population

	Study group (n = 475)	Excluded group (n = 313)	p-value
Age (years)	65.9 ± 10.2	65.3 ± 11.0	0.43
Sex (male)	330 (69.4)	221 (70.6)	0.73
BMI (kg/m^2)	23.9 ± 3.2	24.6 ± 3.4	0.004
**Clinical presentation**			
Myocardial infarction	143 (30.1)	72 (23.0)	0.029
Unstable angina	146 (30.7)	108 (34.5)	0.27
Stable angina	163 (34.3)	105 (33.5)	0.82
**Past medical history**			
Previous CAD	10 (2.1)	10 (3.1)	0.34
Hypertension	308 (64.8)	208 (66.4)	0.64
Diabetes	213 (44.8)	115 (36.7)	0.024
Dyslipidemia	83 (17.4)	55 (17.5)	0.97
Cerebrovascular accident	40 (8.4)	26 (8.3)	0.96
Peripheral artery disease	80 (16.8)	22 (7.0)	<0.001
Previous malignancy	32 (6.7)	22 (7.0)	0.87
Current smoker	163 (34.3)	87 (27.7)	0.05
Frailty (CFS ≥5)	121 (25.4)	59 (18.8)	0.030
Mild to moderate (CFS 5-6)	105 (22.1)	51 (16.2)	
Severe (CFS 7)	16 (3.3)	8 (2.5)	
**Laboratory data**			
Total cholesterol (mmol/L)	4.09 (3.42-4.95)	4.22 (3.54-5.02)	0.29
LDLc (mmol/L)	2.56 (1.89-3.32)	2.75 (1.97-3.39)	0.32
hs-CRP (mg/L)	3.0 (1.0-11.0)	2.0 (0.9-7.9)	0.11
HbA1c (%)	6.52 ± 1.68	6.49 ± 1.27	0.77
CrCl (mL/min)	73.8 ± 31.4	78.2 ± 32.0	0.05
LVEF (%)	53.1 ± 10.3	54.6 ± 10.0	0.05
**Post-PCI medications**			
Aspirin	436 (91.7)	288 (92.0)	0.91
Clopidogrel	417 (87.7)	271 (86.5)	0.62
Beta blocker	229 (48.2)	144 (46.0)	0.54
Calcium channel blocker	150 (31.5)	103 (32.9)	0.70
ACE-inhibitors and ARBs	296 (62.3)	187 (59.7)	0.47
Statins	382 (80.4)	266 (84.9)	0.10
**Procedural profiles**			
Number of treated lesions	1.7 ± 1.0	1.7 ± 1.1	0.65
Number of treated vessels	1.3 ± 0.6	1.3 ± 0.6	0.25
Treated vessels			

Left main	17 (3.5)	10 (3.1)	0.77
LAD	278 (58.5)	187 (59.7)	0.73
LCX	134 (28.2)	111 (35.4)	0.031
RCA	181 (38.1)	108 (34.5)	0.31
Lesion type B2C	447 (94.1)	294 (93.9)	0.92
Multivessel disease	117 (24.6)	92 (29.3)	0.14
Number of inserted stents	1.68 ± 0.94	1.70 ± 0.99	0.84
Average stent diameter (mm)	2.97 ± 0.42	2.91 ± 0.53	0.07
Total stent length (mm)	39.9 ± 26.7	40.0 ± 27.1	0.94
Bare metal stents	10 (2.1)	9 (2.8)	0.49
Drug eluting stents	468 (98.5)	300 (95.8)	0.019
1st generation	93 (19.5)	66 (21.0)	0.61
2nd generation	375 (78.9)	234 (74.7)	0.17

Data are expressed as n (%), mean ± standard deviation (SD) or median (interquartile range). ACE = angiotensin converting enzyme; ARB = angiotensin receptor blocker; BMI = body mass index; CAD = coronary artery disease; CFS = Clinical Frailty Scale; CrCl = creatinine clearance; hs-CRP = high sensitivity C-reactive protein; LDLc = low density lipoprotein cholesterol; LAD = left anterior descending artery; LCX = left circumflex artery; LVEF = left ventricular ejection fraction; PCI = percutaneous coronary intervention; RCA = right coronary artery

Table S7. Kaplan-Meier survival analysis of 3-year clinical outcomes: comparison between included and excluded population

	Study group (n = 475)	Excluded group (n = 313)	Log-rank p-value
All-cause mortality	50 (11.1)	24 (8.1)	0.14
Cardiovascular	17 (3.9)	14 (4.7)	0.61
Non-cardiovascular	33 (7.0)	10 (3.6)	0.018
Non-fatal MI	17 (4.0)	10 (3.4)	0.69
ST-elevation MI	9 (2.1)	6 (2.0)	0.96
Non-ST-elevation MI	8 (1.9)	4 (1.4)	0.59
Repeat revascularization	46 (12.0)	42 (15.0)	0.19
TVR	36 (9.4)	28 (9.9)	0.69
non-TVR	15 (4.0)	15 (5.5)	0.34
MACE	95 (21.8)	64 (21.7)	0.91

Data are expressed as incidence (%). MACE = major adverse cardiovascular event; MI = myocardial infarction; PSM = propensity-score matched; TVR = target vessel revascularization
